# Community Perceptions of Person-Centered Maternity Care in Migori County, Kenya

**DOI:** 10.3389/fgwh.2021.668405

**Published:** 2021-10-08

**Authors:** Osamuedeme Odiase, Beryl Akinyi, Joyceline Kinyua, Patience Afulani

**Affiliations:** ^1^Institute for Global Health Sciences, University of California, San Francisco, San Francisco, CA, United States; ^2^Global Programs for Research and Training, Nairobi, Kenya; ^3^Kenya Medical Research Institute, Nairobi, Kenya; ^4^Department of Epidemiology and Biostatistics, University of California, San Francisco, San Francisco, CA, United States

**Keywords:** maternity care, maternal health, person-centered care, respectful care, perception, Sub-Saharan Africa (SSA), Kenya, respectful maternity care

## Abstract

**Background:** Community perceptions of quality of maternal healthcare services—including Person-centered maternity care (PCMC)—influences the health-seeking behavior of women. Yet few studies have examined this quantitatively. This study aims to examine community perceptions of PCMC and its associated factors.

**Materials and Methods:** We used data from a survey on community perceptions of PCMC in Migori County, Kenya conducted in August 2016. Community members were relatives or friends of women aged 15 to 49 years who gave birth in the 9 wk preceding the survey (*N* = 198). PCMC was measured using a 30-item scale with three sub-scales for dignity and respect, communication and autonomy, and supportive care. PCMC scores were standardized to range from 0 to 100, with higher scores indicative of higher PCMC. Descriptive, bivariate, and multivariate analyses were performed in STATA (version 14).

**Results:** The average total PCMC score was 62 (*SD* = 15.7), with scores of 74, 63, and 53 for dignity and respect, supportive care, and communication and autonomy, respectively. Controlling for other factors, respondents who were employed and literate had higher PCMC perception scores than those who were less literate and unemployed. Respondents who rated their health as very good had higher PCMC perception scores than those who rated their health as poor. Female respondents who previously gave birth at a health facility had lower perceptions of dignity and respect than those with no prior facility birth.

**Conclusion:** The findings imply that community perceptions of PCMC, particularly related to communication and autonomy, are poor. Given the effects of these perceptions on use of maternal health services, there is a need to improve PCMC.

## Introduction

Sub-Saharan Africa bears the heaviest global maternal mortality burden, with the highest maternal mortality ratio (MMR) in 2017 at 534 deaths per 100,000 live births (1). While Kenya's MMR is below the average for Sub-Saharan Africa, its MMR remains alarmingly high at 342 maternal deaths per 100,000 live births (2). It is estimated that a woman in Kenya dies every two h during childbirth (3). Other evidence suggests that 16–33% of maternal deaths could be prevented if delivery was supervised by a skilled birth attendant (SBA) (4, 5). In an effort to reduce Kenya's MMR, the Kenyan Government abolished user fees – beginning in June 2013 – for all maternal health services in public health care facilities (6). Yet, many women still chose to give birth outside of health facilities. In fact, although 96% of women received antenatal care, only 62% of women gave birth in health facilities in 2014 (7, 8).

Several factors contribute to the low utilization of facility-based delivery services, including proximal factors such as perceptions of need, access, and quality of care (9). These proximal factors are shaped by broader socioeconomic and sociocultural factors. For example, one study that examined the factors that influenced a woman's choice of birth attendants in Kenya found that 94.8% of women would choose to seek a SBA at delivery (10). However, the cost of SBAs and women's education level were strongly correlated with a woman's choice to seek a SBA at delivery (10). Another study in Kenya investigating the social barriers to maternity care found that the social influence of mothers deterred women from utilizing maternity care services (11). The study also found that men tended to favor deliveries under the supervision of a SBA, thus adding to the growing body of evidence that male partners serve as a strong social determinant of women's access to maternity services (11).

Person-centered maternity care refers to care that is respectful and responsive to the needs and values of individual women and their families during childbirth (12). Respectful maternity care is core to PCMC and consists of respect for women and their beliefs, culture, and traditions during labor and childbirth (13). PCMC is not only instrumental in improving both maternal and neonatal outcomes, but it has been deemed a universal human right by the World Health Organization (14). The major components of PCMC – communication and autonomy, dignity and respect, and supportive care – play a significant role in the quality of maternal health care (12). According to the World Health Organization, the key dimensions of PCMC are critical for a safer and more positive childbirth experience (15). Despite efforts to improve PCMC, gross disparities still exist, especially among socioeconomically disadvantaged women (12, 16). A study investigated the quality of PCMC in Kenya, Ghana and India and found that women reported poor PCMC, especially in the communication and autonomy domain (12). In particular, 57% of women reported that medical providers never obtained their consent before performing medical procedures (12). Moreover, 16% of women disclosed verbal abuse and 3% of women reported physical abuse, including pinching and slapping (12). The study also found that women of a lower socioeconomic status received lower quality PCMC than women of a higher socioeconomic status (12).

Maternal perceptions of quality of care, especially related to PCMC, is a key determinant in the likelihood that women will seek facility-based care services or SBAs at delivery. A study in Kenya found that nurses' harsh treatments was a major deterrent to seeking health care services at delivery (11). Prior studies have also found that women who experienced inadequate and discourteous care during delivery were significantly less likely to use professional, supervised delivery care for their next delivery; in turn, this increases the risk of maternal and neonatal deaths (17, 18). The negative maternal perceptions toward facility-based care have significant implications for an increased risk of maternal and neonatal deaths.

Maternal perceptions are influenced by their own previous experiences as well as by the experiences of other women in their community. Community perceptions therefore play a key role in women's use of maternity services. Research shows that women who experience poor quality of care discourage other women from utilizing maternal health services (19). Moreover, there is a chain reaction where women's poor perceptions of PCMC contribute to negative community perceptions of quality of care (20–23). In turn, poor community perceptions negatively impact women's maternal health seeking behaviors (20–22). Although a growing body of research has explored maternal perceptions of PCMC, to our knowledge, no quantitative research has empirically investigated community perceptions of PCMC and the factors associated with these perceptions. This study seeks to bridge this gap. Understanding how community members perceive quality of services provided in their community could help pave the way for more effective interventions to improve maternal and neonatal outcomes.

## Materials and Methods

### Sample Selection and Data Collection

The data for this analysis was collected from a larger cross-sectional study that assessed mothers, community members, and healthcare providers' perceptions of PCMC in Migori, County, Kenya. The county is a primarily rural area in Southwestern Kenya and has been described in detail elsewhere (24). Data was collected in August and September 2016.

Data used for this analysis are from family members–spouses, mothers-in-law, and mothers–and friends of women who participated in the mothers' survey: women aged 15 to 49 yr who delivered nine wk prior to recruitment (at home or in healthcare settings). Ten to twelve health units were randomly selected from each of the eight sub-counties in the county, and the family members of the first two women recruited for the mothers' survey were invited to participate in the community survey. About 25 family members were interviewed from each sub-county (range of 16 to 38 depending on sub-county size) for a total of 198 respondents.

The data collectors provided information about the study, including the purpose of the study and the eligibility criteria for participation in the study and obtained informed consent from all respondents. They then conducted the interviews using a structured questionnaire which contained demographic as well as PCMC questions. The interviews were conducted in English or the local languages (Swahili and Luo) at health facilities or in their homes. Ethical approval for the study was obtained from the University of California, San Francisco and the Kenya Medical Research Institute, with additional permissions from the county.

### Variables

#### Dependent (Outcome) Variables

**Figure 1:**
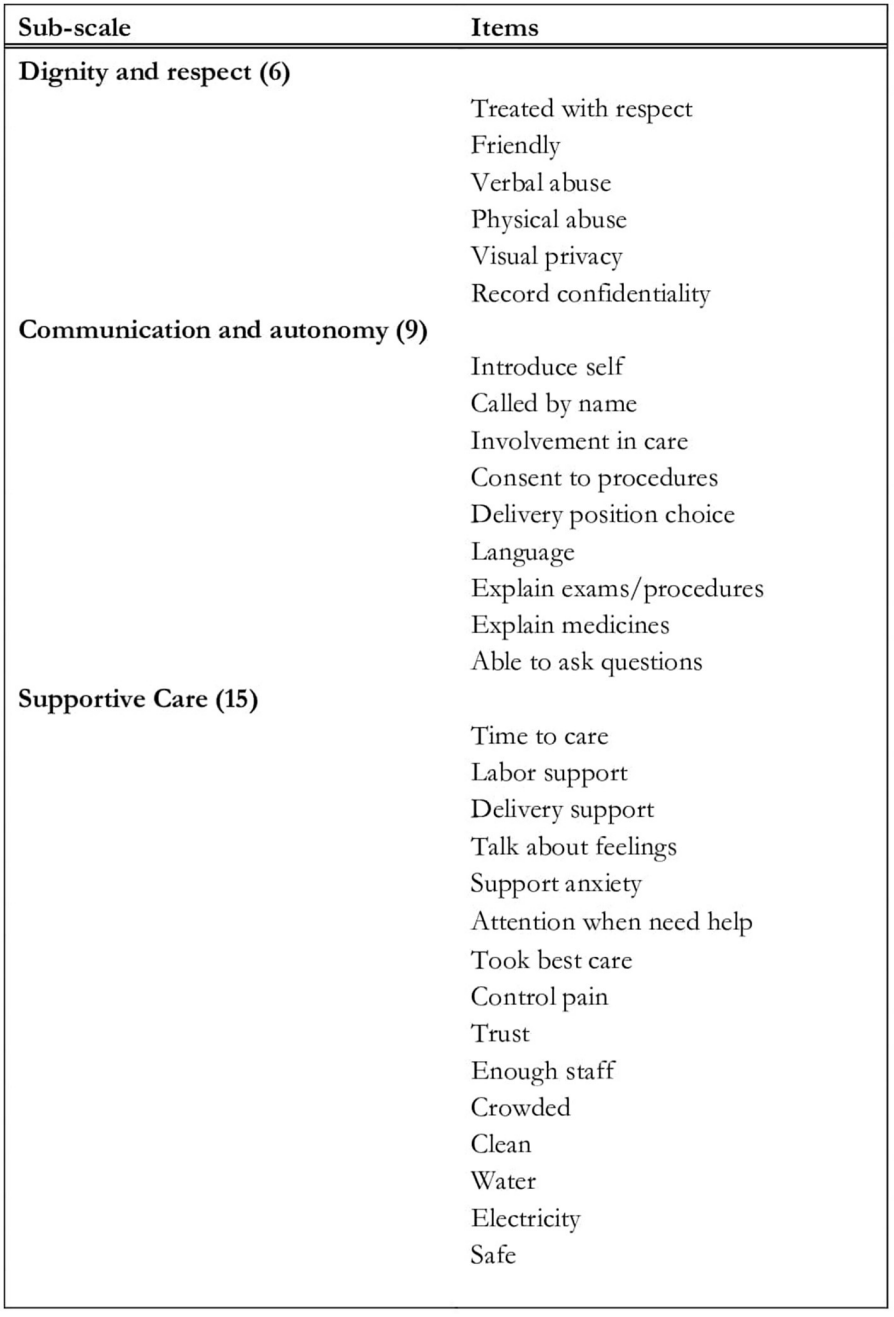
Items from the 30-item PCMC scale by domains.

PCMC was measured with a modified version of the PCMC scale (Figure 1), which was initially developed to measure women's perceptions of PCMC. The original PCMC scale, which was initially validated in Kenya with the mothers' sample, was re-worded to inquire about family members' general perceptions of PCMC. Studies with recently delivered women in Kenya, Ghana, and India shows the original scale has high validity and reliability (25). The PCMC scale has 30 items, with each question having a 4-point frequency response options [i.e., 0-(“no, never”), 1-(“yes, a few times”), 2-(“yes, most of the time”), and 3-(“yes all of the time”)] (24). The responses to the 30 items are summed to generate a summative total PCMC score ranging from zero to 90, where a low PCMC score is indicative of poor PCMC. Scores are also generated for its three sub-scales that measure the perceptions of dignity and respect, communication and autonomy, and supportive care. The dignity and respect sub-scale consists of six questions, which include whether healthcare providers demonstrated respectful care, physically or verbally abused women, and if healthcare providers treated women in a friendly manner, with sub-scale scores ranging from zero to 18. The communication and autonomy sub-scale consists of nine questions, which include whether health care providers obtained consent or explained medical procedures to the women, with scores from zero to 27. Finally, the supportive care sub-scale consists of 15 questions, which include questions about supportive care provided by the providers and healthcare facilities, with scores from zero to 45.

#### Independent (Predictor) Variables

We examined how the following factors may be associated with PCMC: age, sex, literacy, education, employment status, religion, ethnicity, self-rated health, prior facility delivery, number of children and births, and relationship to the woman.

### Statistical Analyses

Analyses were performed using version STATA (version 14). Descriptive statistics were performed to examine the demographic characteristics of respondents and the distribution of PCMC and sub-scale scores. Prior to generating summative scores, negatively worded questions were reverse coded. Additionally, “I don't know” responses were recoded to “2-(“yes, most of the time”).” We however performed sensitivity analysis, in which the “I don't know” responses were recoded to missing. Missing responses in the full PCMC scale and sub-scales were excluded from the main analysis. We also conducted factor analysis to assess psychometric properties of the scale based on this sample. This showed the modified scale has high construct validity with factor analysis yielding 3 factors with eigenvalues greater than 1, but with one dominant factor. Cronbach's alpha was 0.89 representing high internal consistency. To allow for comparison between scores, the PCMC scale and sub-scale scores were standardized to range from zero to 100 by dividing the score by the maximum possible score and multiplying by 100. We then used cross tabulations to express the bivariate distribution of the predictor and outcome variables and used OLS regression to examine statistically significant associations.

We used backward stepwise multivariate regression to select final models to examine the associations between predictor variables on the full PCMC and sub-scale scores. We built the multivariate models by starting with all the potential predictors, and at each step, removing non-significant variables from the regression models until the reduced models that best explained the data were observed. AIC values were used to choose the best models that explained the data. A *p*-value of less than 0.05 was considered statistically significant. Age and sex, although not significant, were included in the final models based on their conceptual importance. We also performed collinearity tests and evaluated model fit to exclude closely related variables (26). Therefore, only variables that were not collinear with other predictor variables and improved the model were included in the full model (26).

## Results

### Univariate Analyses

A summary of sociodemographic statistics of community members can be seen in Table 1. The average age of respondents was about 37 yrs old, and about 40% were under the age of 40. About 60% of respondents were male spouses of women in the mothers' survey, with 26% being mothers or mothers-in-law. Around 83% of the female respondents had previously given birth in a health facility. The average number of children and births was two. Approximately 14% reported poor or fair health status. Regarding education, about 30% possessed a primary level education or less and a lower literacy level. About 61% of respondents were unemployed. Almost all of the respondents were Christians. Close to 70% belonged to the Luo Ethnicity.

**Table 1:** Univariate sociodemographic distribution of predictor variables.

	***No*.**	***%***
**Total No**	**198**	**100.0**
**Age**		
20 to 29 years	46	23.2
30 to 39 years	80	40.5
40 to 49 years	46	23.2
50 to 69 years	26	13.1
**Sex**		
Male	123	62.1
Female	75	37.9
**Relation to woman**		
Partner/Husband	116	58.6
Mother/Mother-in-law	52	26.3
Sister-in-law/Friend-neighbor/Other	30	15.1
**Education**		
No school/Primary	52.0	26.3
Post-primary/vocational/Secondary	69.0	34.8
College or above	77.0	38.9
**Literacy: reading**		
No, cannot read; Yes, but with some difficulty	56.0	28.3
Yes, very well	142.0	71.7
**Literacy: writing**		
No, cannot write; Yes, but with some difficulty	58.0	29.3
Yes, very well	140.0	70.7
**Work for pay**		
No	120.0	60.6
Yes	78.0	39.4
**Religion** Catholic/Methodist/Presbyterian/Anglican	69.0	34.9
Protestant/Pentecostal/Other Christian	46.0	23.2
Seventh Day Adventist	75.0	37.9
Muslim/Other Religion/ No Religion	8.0	4.04
**Tribe**		
Luo	134.0	67.7
Other	64.0	32.3
**Number of births**		
0 to 2	54	27.3
3 to 5	74	37.4
More than 5	70	35.3
**Number of children**		
0 to 2	60	30.3
3 to 5	83	41.9
More than 5	55	27.8
**Past facility delivery (women)**		
No	12	17.0
Yes	59	83.0
**Health status**		
Poor or Fair	27	13.6
Good	124	62.6
Very good or excellent	47	23.8

### Distribution of PCMC and Sub-scale Scores

The distribution of the individual PCMC items is shown in Appendix 1. The full PCMC and sub-scales scores are shown in Table 2. The standardized average PCMC score was 62 (*SD* = 15.7). Among the three PCMC sub-scales, dignity and respect had the highest standardized mean score of 74 (*SD* = 19.3), followed by supportive care with a standardized mean score of 63 (*SD* = 15.0), and communication and autonomy with a standardized average score of 53 (*SD* = 21.8). The sensitivity analysis in which the “I don't know” responses were recoded to missing values resulted in slightly lower full PCMC and subscale scores (standardized scores of 59.1, 64.0, 50.8, and 61.9, respectively for full scale, dignity and respect, communication and autonomy, and supportive care).

**Table 2:** Distribution of Standardized Full PCMC and Sub-scale scores.

**Name of Scale**	***N***	**Score**	***SD***	**Minimum Score**	**Maximum Score**
Full PCMC scale	195	61.9	15.7	13.3	94.4
Dignity and Respect (sub-scale)	198	73.6	19.3	11.1	100.0
Communication and Autonomy (sub-scale)	196	52.8	21.8	0.0	100.0
Supportive Care (sub-scale)	196	62.5	15	17.8	93.3

### Bivariate Analyses

The bivariate statistics of the full PCMC are shown in Table 3 and that for sub-scale scores in Appendix 2. The factors significantly associated with the full PCMC or sub-scale scores in the bivariate analysis were age, sex, relationship to the woman, religion, literacy, work for pay, self-rated health status, and past facility delivery. Respondents older than 49 yrs had lower PCMC perceptions, with, on average, 10 points lower scores on the full PCMC scale and 17 points lower scores on the dignity and respect and communication and autonomy sub-scales than younger respondents (20–29 yrs old). Female respondents scored, on average, five points lower on the full PCMC scale and on the communication and autonomy sub-scale than male respondents. Relationship to the woman was significantly associated with communication and autonomy, where mothers of the women had, on average, eight points lower scores on PCMC than male spouses of the women. Respondents with a lower literacy level scored, on average, 11 points lower on the full PCMC scale than those who were more literate. Also, unemployed respondents scored, on average, eight points lower on PCMC than respondents who were employed. Additionally, respondents who rated their health as poor or fair received significantly lower PCMC (53.7) than those who rated their health as very good or excellent (66.9). Prior birth in a health facility was associated with lower perceptions of dignity and respect.

**Table 3:** Bivariate statistics of predictor variables on the full PCMC score.

	**Full PCMC score**					
	**Crosstabs**		**OLS regression**			
**Predictor Variables**	***Mean***	***SD***	***Coeff*.**	***[95% CI]***		***P-value***
**Age**						
20 to 29 years (Reference Group)	60.8	15.9	0.0			
30 to 39 years	66.0	13.2	5.2	−0.3	11	0.0650
40 to 49 years	62.4	17.9	1.6	−4.7	7.8	0.626
50 to 69 years	50.6	13.6	−10	−18	−3.0	0.00600
**Sex**						
Female (Reference Group)	58.9	15.1	0.0			
Male	63.8	15.8	4.8	0.3	9.4	0.0370
**Relation to woman**						
Partner/Husband (Reference Group)	63.6	16.0	0.0			
Mother/ Mother-in-law	59.1	16.2	−4.5	−9.7	0.7	0.0930
Sister-in-law/Friend-neighbor/Other	60.5	13.2	−3.0	−9.4	3.3	0.344
**Education**						
No school/Primary (Reference Group)	60.2	18.2	0.0			
Post-primary/vocational/Secondary	60.0	15.3	−0.2	−5.9	5.5	0.947
College or above	64.9	13.9	4.7	−0.9	10	0.0970
**Literacy: reading**						
No, cannot read; Yes, but with some difficulty (Reference Group)	54.2	14.7	0.0			
Yes, very well	64.9	15.2	10.0	5.7	15	0.000
**Literacy: writing**						
No, cannot write; Yes, but with some difficulty (Reference Group)	55.4	14.8	0.0			
Yes, very well	64.7	15.3	9.3	4.6	14	0.000
**Income**						
Less than 5000 Ksh ($50 USD) (Reference Group)	69.9	14.5	0.0			
More than 5000 Ksh ($50 USD)	63.8	14.1	−6.1	−13	0.9	0.0870
Refused to Answer	68.1	12.7	−11.0	−17	−4.9	0.000
**Work for Pay**						
No	58.8	15.9	0.0			
Yes	66.6	14.4	7.8	3.4	12	0.00100
**Religion**						
Catholic/Methodist/Presbyterian/Anglican (Reference Group)	58.5	14.5	0.0			
Protestant/Pentecostal/Other Christian	64.5	17.0	6.0	0.8	11	0.0240
Seventh Day Adventist	63.1	14.4	4.6	−1.4	11	0.131
Muslim/Other Religion/ No Religion	60.1	17.2	1.6	−9.9	13	0.782
**Tribe**						
Luo (Reference Group)	61.5	13.8	0.0			
Other	62.8	19.1	1.3	−3.4	6.1	0.578
**Number of births**						
0 to 2 (Reference Group)	62.8	12.1	0.0			
3 to 5	64.9	17.0	2.2	−3.4	7.7	0.442
more than 5	58.2	16.2	−4.6	−10	1.0	0.106
**Number of children**						
0 to 2 (Reference Group)	62.1	14.0	0.0			
3 to 5	64.0	16.8	1.8	−3.4	7.1	0.492
more than 5	58.7	15.7	2.9	−9.2	2.3	0.240
**Past facility delivery**						
No (Reference Group)	63.4	17.2	0.0			
Yes	57.9	15.0	−5.6	−15	4.2	0.260
unknown	63.7	15.7	0.3	−9.0	9.6	0.957
**Current health status**						
Poor and Fair (Reference Group)	53.7	13.9	0.0			
Good	61.9	16.6	8.2	1.7	15	0.0130
Very good and excellent	66.9	12.2	13.0	5.9	20	0.000

### Multivariate Analyses

Table 4 shows the final multivariate models of the full PCMC and sub-scale scores. After accounting for other covariates, respondents who were employed and literate had significantly higher PCMC scores. Literate respondents had about 10 more points on communication and autonomy and 7 more points on supportive care than less literate respondents. Additionally, employed respondents scored, on average, 10 and 7 points higher on communication and autonomy and supportive care, respectively. Respondents in the 30 to 39 age group scored, on average, five points higher on supportive care than respondents in the 20 to 29 age group. On average, respondents who identified as Catholic, Methodist, Presbyterian or Anglican had significantly lower communication and autonomy sub-scale scores than those who identified as Protestant or Pentecostal. After controlling for other factors, Luo respondents had significantly lower supportive care sub-scores than respondents from other ethnicities. Respondents who rated their health as very good or excellent had higher PCMC perceptions, and scored, on average, fifteen points higher on communication and autonomy than respondents with poor or fair self-rated health.

**Table 4:** Multivariate linear regression analysis of select predictors on the full PCMC and sub-scale scores.

	**Full PCMC score**			**Dignity and Respect Sub-score**			**Communication and Autonomy Sub-score**			**Supportive Care** **Sub-score**		
	***Coeff*.**	***[95% CI]***		***Coeff*.**	***[95% CI]***		***Coeff*.**	***[95% CI]***		***Coeff*.**	***[95% CI]***	
**Age**												
20 to 29 years				0			0			0		
30 to 39 years	4.61	−0.78	10	5.90	−0.9	13	2.19	−4.9	9.3	5.45^*^	0.16	11
40 to 49 years	5.53	−1.0	12	3.00	−4.8	11	5.96	−2.7	15	3.65	−2.6	9.9
50 to 70 years	−2.00	−11.0	6.5	−10.0	−20.0	0.26	−4.59	−16	6.7	0.469	−7.6	8.5
**Sex**												
Female				0								
Male	−0.83	−6	4.1	−1.90	−8.0	4.1	0.31	−6.2	6.8	−0.88	−5.7	3.9
**Literacy (reading)**												
No, cannot read; Yes, but with some difficulty		0.0	0.0					0.0	0.0		0.0	0.0
Yes, very well	7.63^*^	1	14	2	−5.4	9.4	9.6^**^	1.4	18	6.91^**^	1.1	13
**Work for pay**												
No		0.0	0.0									
Yes	5.81^**^	1.5	10	6.3^**^	0.9	12	9.96^***^	4.3	16	3.82	−0.4	8.1
**Tribe**												
Luo (Reference Group)		0.0	0.0		0.0	0.0					0.0	0.0
Other	2.63	−2.1	7.4	4.30	−1.40	10				4.72^*^	0.28	9.1
**Religion**												
Catholic/Methodist/Presbyterian/Anglican		0.0	0.0					0.0	0.0			
Protestant/Pentecostal/Other Christian	3.60	−1.40	8.6				8.73^***^	2.3	15			
Seventh Day Adventist	1.80	−3.8	7.4				5.41	−2.0	13			
Muslim/Other Religion/ No Religion	6.47	−5.3	18				8.90	−6.7	25			
**Current health status**												
Poor and Fair		0.0	0.0					0.0	0.0		0.0	0.0
Good	3.94	−2.8	11				4.30	−4.7	13	3.8	−2.5	10
Very good and excellent	8.22^*^	0.6	16				14.9^***^	4.9	25	4.93	−2.3	12
N	195			198			196			196		
R-squared	0.209			0.118			0.269			0.137		
AIC	1,606.77			1,723.34			1,725.45			1,607.42		
BIC	1,649.32			1,749.64			1,764.79			1,640.20		

*** *p < 0.001*,

** *p < 0.01*,

* *p < 0.05*.

## Discussion

The aim of this study was to understand community perceptions of PCMC and the associated sociodemographic factors. We found that community perceptions of PCMC was low, with the lowest perceptions related to communication and autonomy. The factors associated with PCMC among relatives and friends of women who had recently given birth were literacy, employment status, ethnicity, and self-rated health status. Literate and employed community members, and those who rated their health as very good or excellent had significantly higher perceptions of PCMC than low literate and unemployed community members, and those who had poor or fair self-rated health. Female community members who had previously given birth at a health facility had significantly lower perceptions of dignity and respect than women who had not previously given birth at a health facility in the bivariate analysis.

Though there is a dearth of quantitative research on community perceptions on the quality of maternal care, it has been explored by a few qualitative studies. Similar with other studies, we find that community members have poor perceptions of quality of maternal care (27, 28). A possible reason for these poor perceptions is the perceived lack of emotional support women receive from healthcare providers (29). Furthermore, these community members may witness their female relatives being unable to effectively express or advocate for themselves, thus contributing to the poorer perceptions of communication and autonomy (28). We also find that less literate community members have poorer perceptions of supportive care (27, 28). One interpretation of this finding relates to the idea of hierarchical social relations (28). Given that literacy is closely related to one's family social status, it could be that community members with a lower literacy level have poorer perceptions of supportive care because they witness the social stigma their female relatives experience within the healthcare system (28, 30). Low literate community members are also likely to have female relatives who possess a lower level of awareness and knowledge of maternal healthcare services (31) which in addition to low perceptions of PCMC, will deter them from using services (9).

Similar to previous studies that have examined the relationship between self-perceived health and perceptions of the quality of healthcare services, we find that people who rated their health as good had more positive experiences with the healthcare system (32–35). Community members who rated their health as very good may have positive experiences with the healthcare system because of their lower interaction with the health system, which they project onto their perceptions of PCMC. Conversely, community members who rated their health as poor may have negative experiences with the healthcare system because of more frequent interactions with the health systems and thus project their negative experiences onto their perceptions of PCMC. A possible reason for why prior birth at a health facility was found to be significantly associated with lower dignity and respect is that women who previously gave birth in a health facility may have experienced mistreatment, neglect, harassment and abuse (18, 36–38). Consequently, the prior mistreatment of these women affected their perceptions of the quality of dignity and respect that their female relatives received during childbirth (18, 39).

The findings on community perceptions of PCMC are also consistent with prior studies on maternal perceptions of PCMC—particularly findings from the related survey on maternal perceptions of PCMC that were collected from recently delivered women as part of the same study (12, 23, 26, 40). The average PCMC score from the survey with women in the same setting was 65.5 compared to 61.9 from the survey with community members, with the lowest scores also in communication and autonomy. The women reported instances of healthcare providers seldomly introducing themselves, allowing them to make decisions, or permitting them to have a birth companion during childbirth (12). Poorer community perceptions of PCMC could thus be due to community members' knowledge of the poor quality of care that their female relatives received: they may have observed this while accompanying their relatives or heard female relatives complain about their care. Studies with women who have recently given birth also show that the most socioeconomically disadvantaged women received lower quality PCMC (12, 26). Given that family members may occupy a similar socioeconomic status, unemployed and less literate community members may perceive a poorer quality of PCMC because of the poorer quality of care their female relatives receive (41, 42). The poorer perceptions of supportive care among Luo community members is also consistent with Luo women's experiences of discriminatory treatment and non-supportive care (23).

### Strengths and Limitations

The study has some limitations. The first limitation is social desirability bias: community members may have given socially acceptable responses with potential under-reporting of socially undesirable behaviors, such as physical or verbal abuse. Consequently, social desirability bias may have contributed to inflated PCMC scores. Additionally, recoding the “I do not know” responses to the highest category potentially overestimated the PCMC scores. This suggests that the perceptions of PCMC are poorer than we found, highlighting the need to address it. The second limitation is generalizability. Given that this study was performed in a rural area, the findings may not be generalizable to urban areas. However, a major strength of this study is the use of a quantitative measure adapted from a validated scale with standardized questions, which facilitates balanced and unbiased responses. Additionally, the PCMC scale's high reliability and validity in prior studies give credence to our findings (12, 25, 26). In addition, this is one of the few studies that has examined factors associated with community perceptions of PCMC, thus adding a valuable contribution to the PCMC literature.

### Conclusions

To our knowledge, this is the first study to quantitatively assess community perceptions of PCMC. This study revealed that community perceptions of PCMC were significantly lacking, particularly related to communication and autonomy. In addition, literacy, employment status, ethnicity, prior birth at a health facility, and self-rated health were significant predictors of PCMC. Our findings have important implications. First, the poor community perceptions of PCMC could deter women from utilizing maternal healthcare services. Furthermore, the disparities in community members' poor perceptions of PCMC may contribute to existing disparities in the areas of maternal healthcare service utilization, maternal morbidity and mortality, and neonatal mortality. Therefore, it is imperative that future research explore the drivers of community members' poor perceptions of PCMC and develop interventions to address them.

Second, the poorer perceptions of PCMC among community members who rated their health as poor, and women who had previously given birth at a health facility could be due to previous negative experiences within the healthcare system. This is consequential because a history of negative experiences within the healthcare system can decrease future utilization of maternal healthcare services and therefore, increase the likelihood of morbidity and mortality during childbirth. Interventions should focus on improving the quality of PCMC that women receive to increase maternal healthcare service utilization and improve maternal and neonatal health outcomes. Healthcare personnel trainings that emphasize the importance of developing interpersonal relationships between women and their healthcare providers and respecting the cultural beliefs, traditions, norms and autonomy of women and their families should be prioritized.

## Data Availability Statement

The original contributions presented in the study are included in the article/Supplementary Material, further inquiries can be directed to the corresponding author/s.

## Ethics Statement

The studies involving human participants were reviewed and approved by University of California San Francisco Institutional Review Board and Kenya Medical Research Institute Scientific and Ethics Review Unit. The patients/participants provided their written informed consent to participate in this study.

## Author Contributions

OO developed the analysis plan, conducted the analysis, and drafted the manuscript. BA and JK supported the data collection and provided feedback on the manuscript. PA is PI of the study and served as a mentor to OO, guided her on the project and analysis, and supported writing of the manuscript. All authors contributed to the article and approved the submitted version.

## Funding

The study was funded by the University of California, San Francisco PTBi Transdisciplinary Post-doctoral Fellowship through the Bill and Melinda Gates Foundation (Grant Number INV-007991) and Marc and Lynne Benioff. The funders were not involved in the study design, collection, analysis, interpretation of data, the writing of this article or the decision to submit it for publication.

## Conflict of Interest

The authors declare that the research was conducted in the absence of any commercial or financial relationships that could be construed as a potential conflict of interest.

## Publisher's Note

All claims expressed in this article are solely those of the authors and do not necessarily represent those of their affiliated organizations, or those of the publisher, the editors and the reviewers. Any product that may be evaluated in this article, or claim that may be made by its manufacturer, is not guaranteed or endorsed by the publisher.

## Abbreviations

PCMC: Person-Centered Maternity Care
MMR: Maternal Mortality Ratio
SBA: Skilled Birth Attendant.
